# Case Report: Ileo-Ileal Intussusception Secondary to Inflammatory Fibroid Polyp: A Rare Cause of Intestinal Obstruction

**DOI:** 10.3389/fsurg.2022.876396

**Published:** 2022-04-15

**Authors:** Claudio Guerci, Francesco Colombo, Gloria Goi, Pietro Zerbi, Barbara Pirrò, Piergiorgio Danelli

**Affiliations:** ^1^Department of General Surgery, Department of Biomedical and Clinical Sciences “Luigi Sacco”, “Luigi Sacco” University Hospital, Università degli Studi di Milano, Milan, Italy; ^2^Pathology Unit, Department of Biomedical and Clinical Sciences “L. Sacco”, “Luigi Sacco” University Hospital, Università degli Studi di Milano, Milan, Italy; ^3^Department of Radiology, “Luigi Sacco” University Hospital, Università degli Studi di Milano, Milan, Italy

**Keywords:** intussusception, fibroid polyp, surgery, histology, IFP, laparoscopy

## Abstract

**Introduction:**

Intussusception is a telescoping of a bowel segment into another and it can be a surgical urgency. Most adult intussusceptions arise from a lead point which can be benign or malignant. For this reason, intussusception in adults should undergo surgery. Here we describe a case of ileal inflammatory fibroid polyp (IFP), presenting with ileo-ileal intussusception and obstruction.

**Case report:**

A 54-year-old Caucasian woman presented for acute abdominal pain. A radiography and a CT of the abdomen were performed, which showed signs of occlusion due to an ileo-ileal intussusception. An urgent laparoscopy was performed, the intussusception was extracorporeally reduced, the ileal segment involved was resected, and an ileo-ileal anastomosis was performed. The intussusception seemed to be caused by a 3-cm intra-mural lesion.

**Discussion:**

Intussusception is a surgical concern. While most cases are idiopathic in children, 90% of adult intussusceptions are caused by underlying diseases. Therefore, intussusception in adults should undergo surgery. Radiology is necessary for the diagnosis: the CT scan helps localizing the lesion and shows pathognomonic signs. This case report analyzes an intussusception caused by an inflammatory fibroid polyp. Accurate diagnosis of IFP is only possible with histopathological examination, helped by immunohistochemistry. The differential diagnosis is important because some lesions are malignant.

**Conclusion:**

We reported a case of intussusception caused by an IFP. The diagnosis was made with a CT scan together with intraoperative findings and histopathological examination, which excluded potential differential diagnoses. The patient underwent an explorative laparoscopy, with an ileal resection and anastomosis. Due to the risk of malignancy, surgery is mandatory.

## Introduction

Intussusception (or invagination) is a telescoping of a bowel loop into the lumen of an adjacent bowel segment. It occurs when a more proximal portion of the bowel (intussusceptum) invaginates into the more distal bowel (intussuscipiens). The pathogenesis seems to be related to an altered bowel peristalsis at the intraluminal lesion, which becomes a lead point for the intussusception (1). This situation prompts venous congestion and tissue edema, it compromises peristalsis and bowel transit. If untreated, intussusception can lead to ischemia, necrosis, and perforation. Intussusception in children is more common than in adults (2) and it is typically due to a benign condition. Conversely, invagination in adults is an extremely rare condition (5% of all intussusceptions). Only 1% of all cases of adulthood bowel obstruction is due to intussusception (3). Besides, intussusception in children tends to be transient and can be treated conservatively, whilst in adults it frequently causes an acute abdomen and should undergo surgery, because of possible underlying neoplasms, benign or malignant. Actually, in contrast to invaginations in children, 90% of adult intussusceptions are caused by a definite lead point (4). Most adult invaginations arise from the small bowel and this lead point is usually a benign condition, such as strictures, adhesions, foreign bodies, vascular anomalies, lymphoid hyperplasia, trauma, celiac disease, cytomegalovirus colitis, lymphoid hyperplasia secondary to lupus, Henoch-Schönlein purpura, Wiskott-Aldrich syndrome, appendiceal stump, Meckel's diverticulum, benign tumors, and like inflammatory fibroid polyps (IFP) (1). In some cases, the lead point can be a malignant lesion, such as adenocarcinomas, lymphomas (5), metastases, carcinoids, leiomyosarcomas, histiocytomas, and gastrointestinal stromal tumor (GIST).

Here we present a rare case of ileal inflammatory fibroid polyp, presenting with ileo-ileal intussusception, and bowel obstruction. Inflammatory fibroid polyp (IFP) is an uncommon gastrointestinal, benign, pseudotumorous lesion, mostly found in the gastric antrum. The small bowel is the second most common site of origin, where IFPs usually present as intussusception or obstruction (1).

## Case Report

A 54-year-old Caucasian woman presented to our emergency unit for acute pain localized in the left abdominal quadrants, since the day before, associated with nausea. The patient reported no fever, vomit, neither change in bowel habits. The patient had a non-contributory past medical history, apart from a suspected irritable bowel syndrome. She reported no allergies nor drugs taken at home regularly.

Physical examination revealed mild dehydration with minimal abdominal distension and tenderness in the left bottom quadrant. The vital signs including temperature, pulse, blood pressure, and respiratory rate were within normal limits. The blood tests revealed mild hypokalemia, which was intravenously corrected, and analgesics were administered, with mild relief.

**Figure 1:**
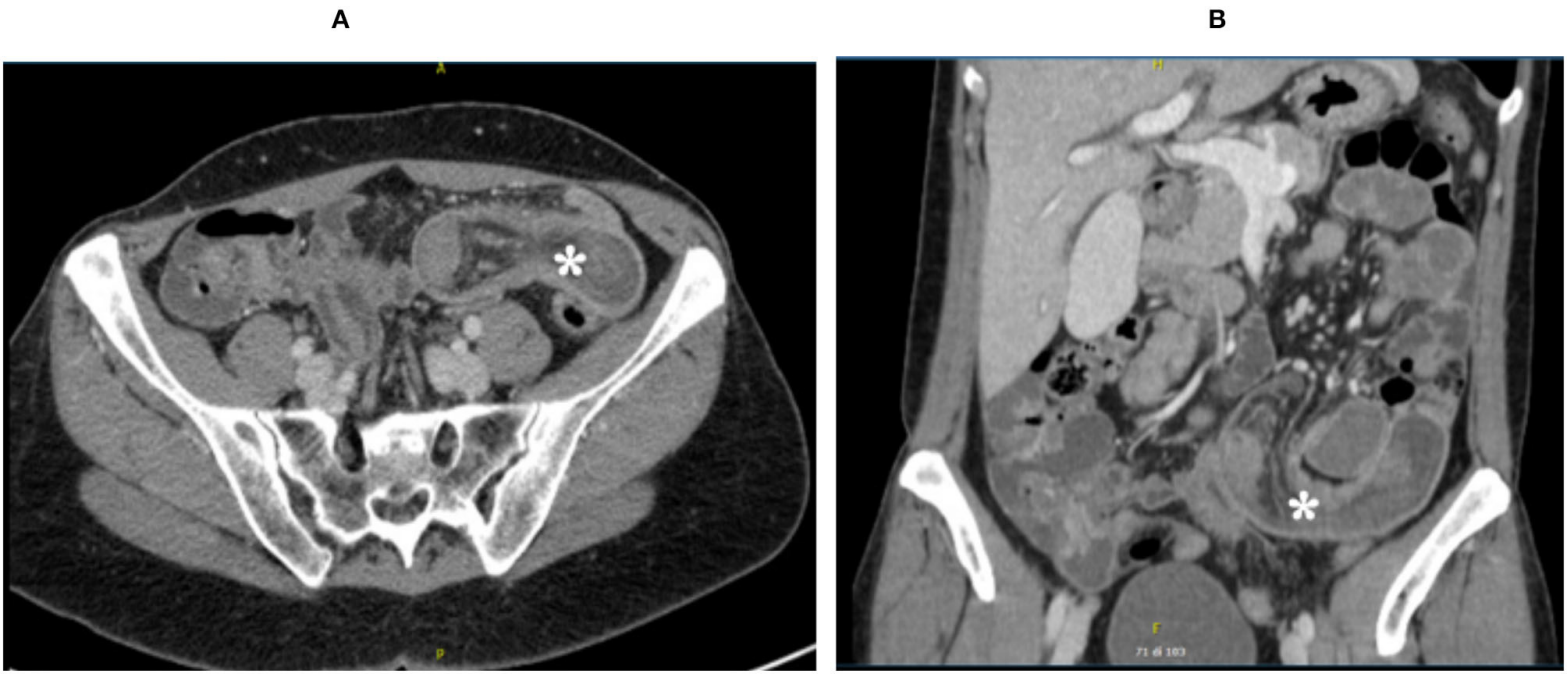
Abdominal CT scan: *indicates the ileo-ileal intussusception. **(A)** CT transverse section. **(B)** CT coronal section.

Plain radiography of the abdomen revealed an isolated air-fluid level in mesogastrium, with diffuse impacted stools. Consequently, an abdominal CT was performed, which showed a wall thickening and hyperemia of a proximal ileal segment, with a typical “target sign” as for ileo-ileal intussusception (Figures 1A,B). The coronal reconstruction showed a “sausage-like” mass; no obvious lead point was identified, nor significant proximal bowel distention was detected. Moreover, some mesenteric lymphadenopathies were observed.

Initially, a conservative treatment was adopted, both because of a relief in symptoms and substantially normal blood test results (white blood cell count: 8.86 × 10^∧^9/L, hemoglobin: 128 g/L, creatinine: 0.65 mg/dL, CRP: 2.1 mg/L, ABG lactate: 0.8 mmol/L).

**Figure 2:**
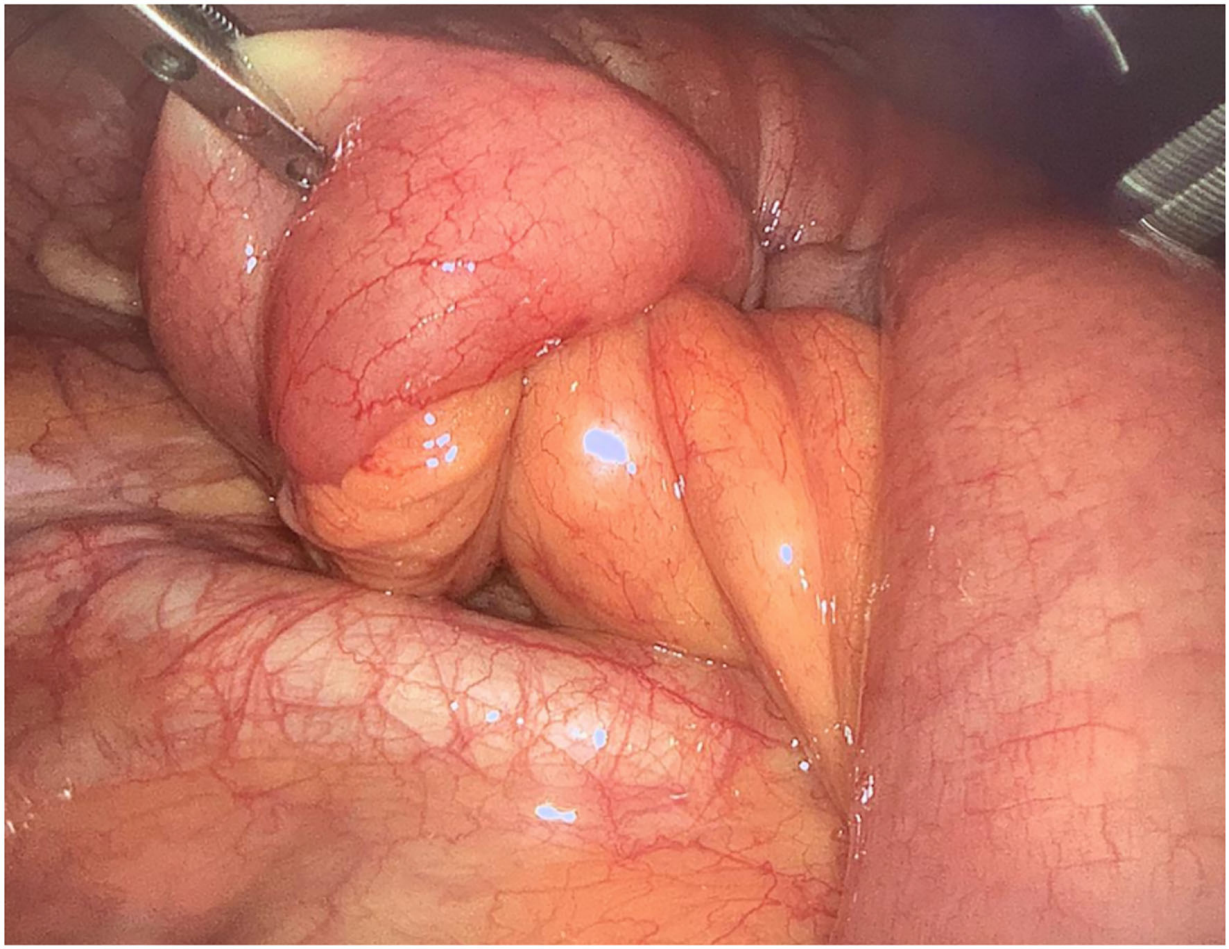
Laparoscopic view of the “lead-point” of the ileo-ileal intussusception.

**Figure 3:**
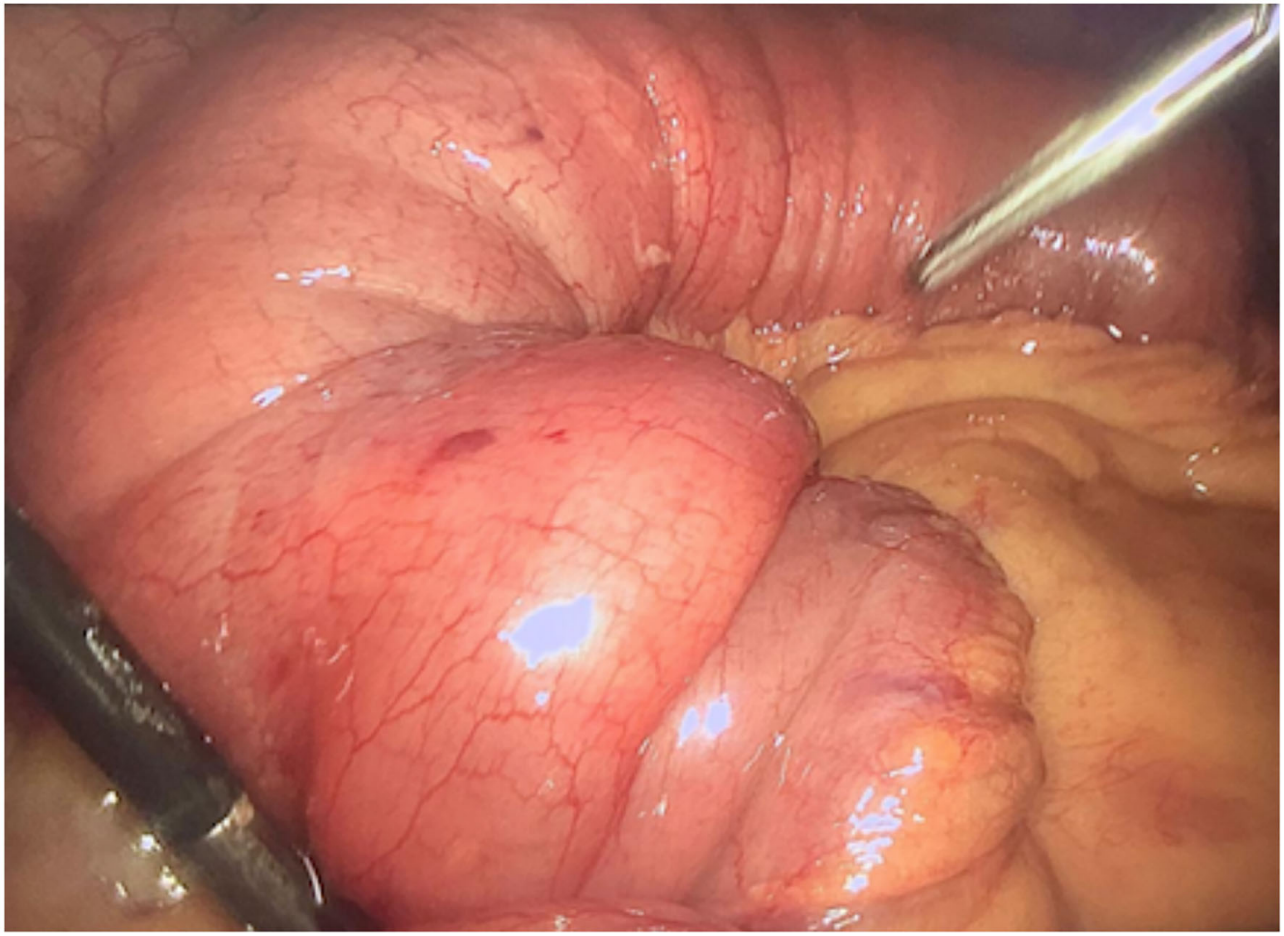
Laparoscopic view of the ileo-ileal invagination: “sausage-shaped” lesion.

Then, after 24 h, the abdominal CT was repeated because of persistence and worsening of symptoms: an increase in the ileum segment involved in the intussusception was documented and free pelvic fluid was observed. As a consequence, an urgent laparoscopy was performed, and a twenty-centimeters-long ileo-ileal invagination was detected (Figures 2, 3). No further lesions were documented by abdominal inspection. An umbilical mini-laparotomy, ~5 cm in length including the umbilical port site, was performed. The affected ileum was easily retracted and therefore the intussusception was reduced. Intraoperatively, the intussusception seemed to be caused by an intra-mural tough lesion with a diameter of about 3 cm. The affected ileum was resected, it was excised with a mesenteric lymph-node, and a hand-sewn side-to-side isoperistaltic anastomosis was performed.

No major complications occurred during the hospital stay. The patient was discharged on the fifth postoperative day.

This case report has been described in accordance with SCARE criteria and PROCESS guidelines (6, 7).

## Histological Findigs

**Figure 4:**
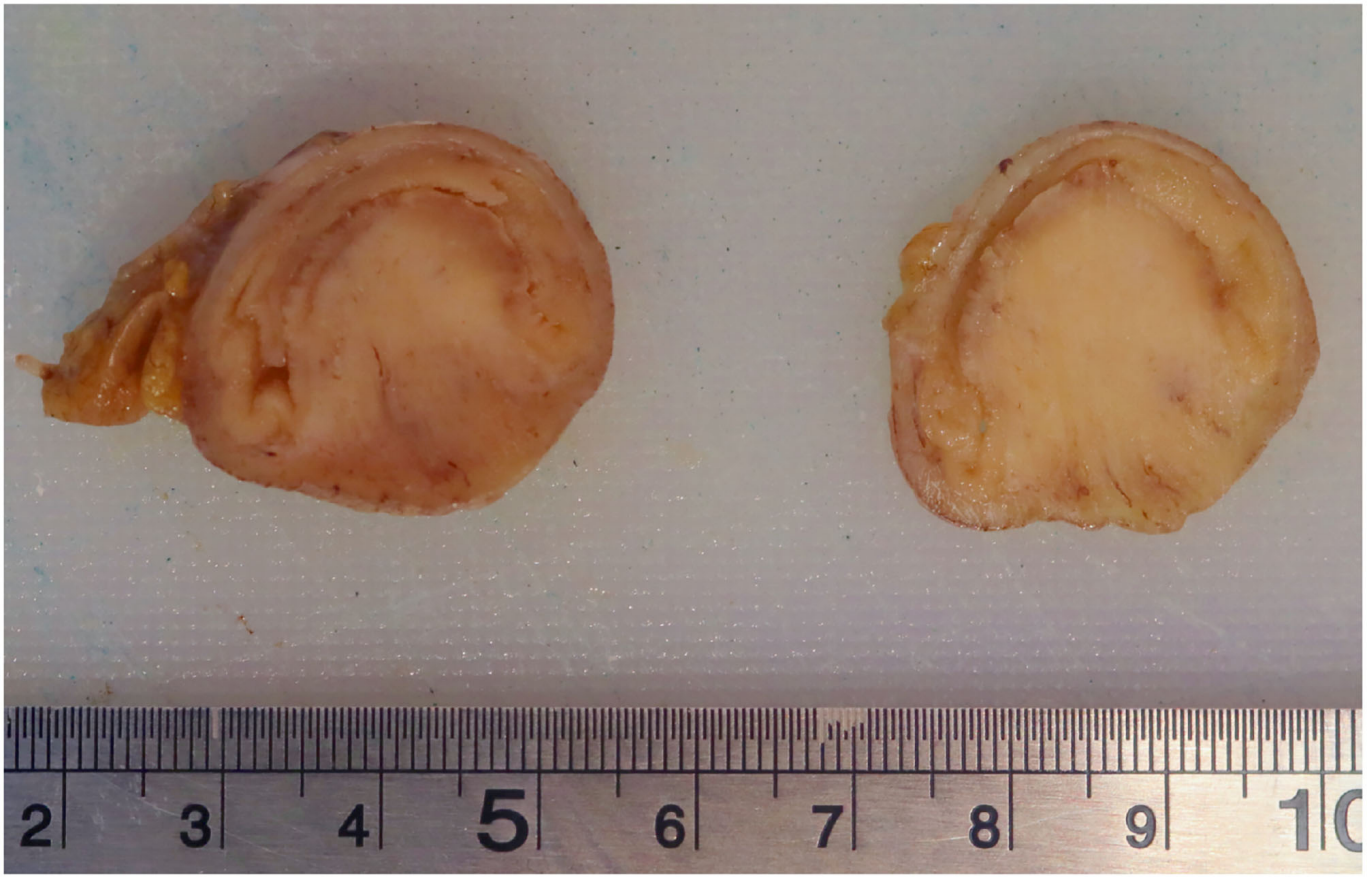
Two cross sections of the ileal tract showing the polypoid lesion.

**Figure 5:**
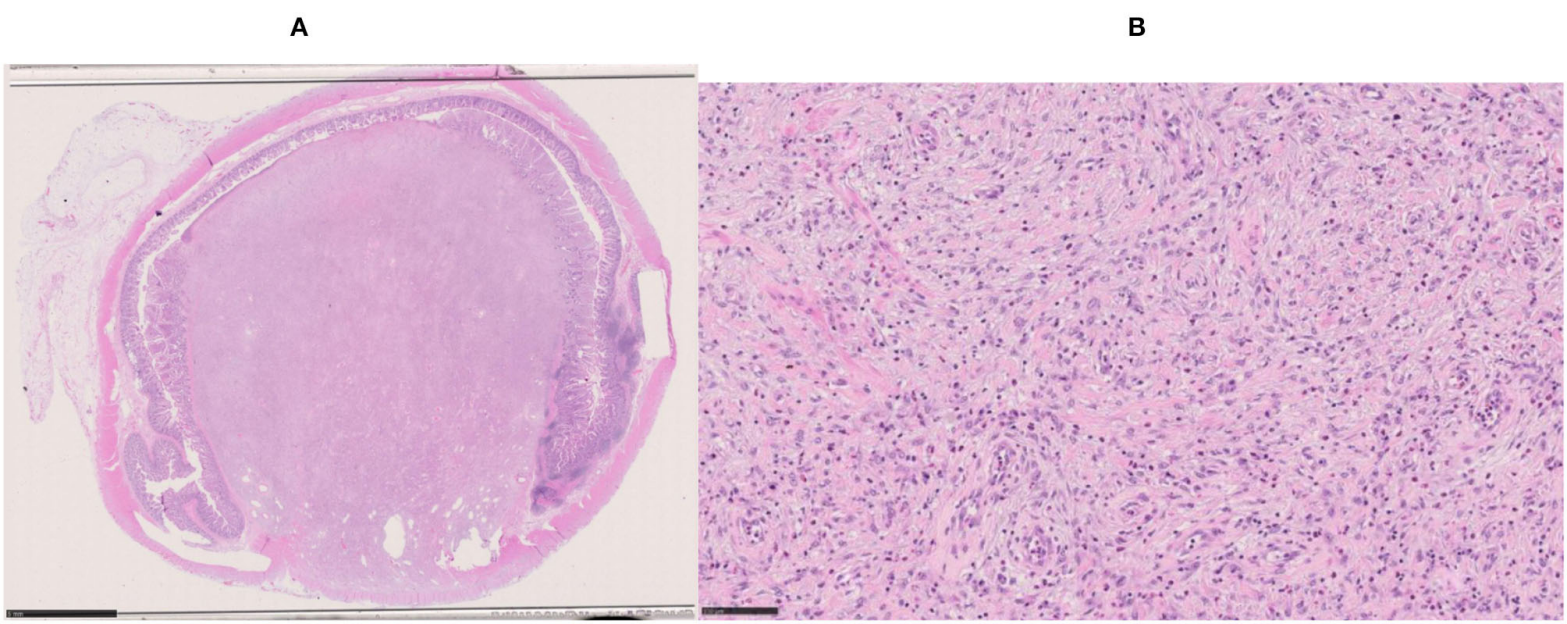
Microscopic view of the inflammatory fibroid polyp. **(A)** Whole-mount section showing complete obstruction of the lumen (Hematoxylin & Eosin, i.o. x1; bar = 5 mm). **(B)** Spindle cells with myofibroblast features admixed with inflammatory cells (Hematoxylin & Eosin, i.o. x20; bar = 100 micrometer).

The seven-centimeters-long small bowel tract was examined. On opening the specimen, a 2.9-cm whitish submucosal polypoid lesion was identified, acting as a lead point of the intussusception. The lesion was coated with ulcerated mucosa and composed of myofibroblastic-like spindle cells admixed with inflammatory cells, including many eosinophils (Figures 4, 5). The immunohistochemical profile of the proliferation was diffusely positive for vimentin, focally positive for smooth muscle actin, and negative for CD34 and for CD117.

## Discussion

Intussusception is a surgical concern. It was firstly described in 1674 by Paul Barbette (8, 9). This condition is described as the telescoping of one bowel segment with its mesenteric fold into an adjoining bowel tract, causing venous congestion and blood supply reduction. Intussusception can occur anywhere along the small and large bowel. Adult intussusception is rarely mentioned, in comparison with that in children. Moreover, while the majority of cases are idiopathic in children, 90% of adult intussusceptions are caused by an underlying disease (4). In children, conservative therapy can be adopted in most cases. It typically consists of non-operative reduction through hydrostatic or/and pneumatic enemas. In case of complications, such as bowel necrosis, perforation, and peritonitis, surgical treatment is indicated, even in children. Adult intussusceptions are rare, with an incidence of 1/1,000,000 cases per year worldwide (10). Almost 90% of adults with intussusception have an underlying lesion, most of them arise from the small bowel, and half of them are malignant (11). Benign conditions can be described by adhesions, strictures, Meckel's diverticulum, inflammatory bowel disease, and benign tumors (lipomas, leiomyomas, and fibroid polyps). Besides, malignant lesions can be metastatic lesions, lymphomas, and adenocarcinomas. Therefore, unlike children, a reduction is not a therapeutic choice, because of the risk of underlying malignant lesions. Intussusception in adults usually occurs with abdominal pain with bowel obstruction signs, but also fever, bowel perforation, bleeding, and abdominal mass palpation could be frequent.

With concern to diagnosis, blood tests can reveal a non-specific increase in the inflammation indexes. Radiology helps in the differential diagnosis. Ultrasonography is cheap and useful, especially if a palpable mass is found, but in most cases, intussusception is better diagnosed with computed tomography (CT). CT scan shows a peculiar sign, described either as “bulls-eye,” “target,” or “sausage-shaped” lesion. This pathognomonic concentric double-ring sign can be identified at coronal and axial view. CT also gives important information about the lesion's location, its nature, its relationship to surrounding organs, and the lymph-node involvement.

This case report analyzes a rare circumstance of intussusception caused by a fibroid polyp. Less than 100 cases of intussusception secondary to ileal polyp are described in the literature (12). An inflammatory fibroid polyp (IFP), also called Vanek's tumor, is a benign submucosal tumor frequently localized in the stomach, especially in the antrum, but it can occur throughout the gastrointestinal tract (13). An IFP was described for the first time in 1949 by Vanek and can also be called eosinophilic granuloma (14). Cases of IFP are reported between 2 and 90 years of age, even if they usually present during the sixth or the seventh decade. Occasionally an IFP can become a lead point for intussusception. Basing on a brief advisory literature review, the median age of the patients suffering from intussusceptions caused by an IFP is about 55. This is probably because intussusception comes with serious signs and symptoms; otherwise, a benign tumor would remain undiagnosed. An IFP is commonly associated with a mutation of the 12th exon of the PDGRF-A gene (15).

IFPs are usually asymptomatic and can be identified during endoscopic procedures and laparoscopies or laparotomies. When symptomatic, the clinical manifestation depends on the location and size of the tumor. Abdominal pain is the most common symptom in patients with lesions in the stomach. Other symptoms (diarrhea, vomiting, tenesmus, alteration in bowel habit) frequency is low (16). The preoperative diagnosis of intussusception is controversial. Abdominal X-ray examination is usually the first diagnostic tool used, because of the obstructive symptoms. Once diagnosis of intestinal obstruction is made, the primary imaging modality of choice is ultrasound imaging, with a sensitivity of 98% and specificity of 88%. However, because of bowel gas interposition and the risk of malignancy, CT scan with contrast is often used (16). Anyway, because of the lack of distinctive radiological features of fibroid polyps, accurate diagnosis of IFP on CT scan is difficult and is only possible with histopathological examination of the anatomical specimen.

Grossly, IFP can be polypoidal (17) or sessile varying in size from 0.2 to 12 centimeters with an average reported size of 4 cm. We reported a 2.9-centimeter-wide lesion acting as a lead point of the intussusception. They arise from the submucosa and project into the bowel lumen. The mucosal surface is usually ulcerated and pale.

Histologically, IFP is composed of an admixture of blood vessels edematous connective tissue, a marked cellular infiltrate which may contain fibroblasts and eosinophils. Some authors reported a sparsely cellular proliferation of spindle cells with fibromyxoid background and copious eosinophils (17), other authors reported the presence of plasma cells and some lymphocytes, in addition to eosinophils (18). The histopathologic sample described in this case report was characterized by an ulcerated mucosa, overlying a submucosal tissue rich in myofibroblastic-like spindle cells.

The IFP immunohistochemical profile can vary. Vimentin, and CD34 can be positive, while SMA, ALK1, CD117 (c-kit), S100, beta-catenin, and desmin are usually negative (18). Negativity for CD 117, CD34, and smooth muscle actin is also testified by Gara et al. (17). On the other side, CD34 was positive in a case report by Feldis et al. (19) and Forasté-Enrìquez et al. (20), while in our case CD34 was negative, as well as CD117. Additionally, our patient presented a positivity for vimentin and focal positivity for smooth muscle actin.

The macroscopic differential diagnosis can be suggested by some peculiarities and epidemiological information (19). For example, adenomatous polyps are more common, but usually smaller, while lipomas can be distinguished on a radiological basis, because of the presence of fat. Lymphomas are more common and usually appear as voluminous endoluminal tumors. A gastrointestinal stromal tumor (GIST) has a similar appearance to IFP, but it usually shows irregular margins, heterogeneous features, and partial extra-luminal development.

Inflammatory myofibroblastic tumor (IMT) and GIST are the main microscopic differential diagnoses (20). IFP shows more eosinophils, fibrosis, and fewer lymphocytes than IMT; in IFP, these cells arise from the submucosal layer, without invasion of the serosa and the muscular layer, which are usually invaded by the IMT cells. The immunohistochemistry as well can help differentiate: ALK1, smooth muscle actin, and occasionally CD117 are usually expressed by the IMT, but not by IFP (which occasionally expresses smooth muscle actin and CD34). It is important to distinguish between IFP and IMT because the IFP does not have a recurrence, while the IMT tends to recur (21). Immunohistochemistry can differentiate between IFP and GIST: both can occasionally express CD34, while only GIST is positive for CD117 (19).

The appropriate management of adult intussusception is controversial, with the debate focusing on mostly on the issue on primary resection vs. reduction followed by more limited resection. Reduction by surgery may theoretically allow more limited resections; however, the risk of seeding or venous dissemination during manipulation of a malignant lesion should be considered (1). In any case, intussusception in adults requires surgery, because of the low accuracy of the imaging and for the risk of malignancy.

## Conclusion

Adult intussusception is a rare entity, and it is usually caused by a lead point. We reported a case of intussusception caused by an inflammatory fibroid polyp in an adult woman. The diagnosis was made with a preoperative CT scan together with intraoperative findings and histopathological examination, which excluded potential differential diagnoses. The patient underwent an explorative laparoscopy, with an extracorporeal resection and ileo-ileal anastomosis. Due to the risk of malignancy, surgery is the best therapeutic chance for intussusception in adults.

## Data Availability Statement

The raw data supporting the conclusions of this article will be made available by the authors, without undue reservation.

## Ethics Statement

Ethical review and approval was not required for the study on human participants in accordance with the local legislation and institutional requirements. The patients/participants provided their written informed consent to participate in this study. Written informed consent was obtained from the individual(s) for the publication of any potentially identifiable images or data included in this article.

## Author Contributions

CG: conception and design of publication, collection of case report data, and writing the manuscript. FC: linguistic check and review the manuscript for important intellectual content. GG: bibliography check and review the manuscript for important intellectual content. PZ: providing pathology images and writing the manuscript. BP: providing and commenting radiological imaging. PD: supervising the case report. All authors contributed to the article and approved the submitted version.

## Conflict of Interest

The authors declare that the research was conducted in the absence of any commercial or financial relationships that could be construed as a potential conflict of interest.

## Publisher's Note

All claims expressed in this article are solely those of the authors and do not necessarily represent those of their affiliated organizations, or those of the publisher, the editors and the reviewers. Any product that may be evaluated in this article, or claim that may be made by its manufacturer, is not guaranteed or endorsed by the publisher.
